# Altered gene expression patterns of innate and adaptive immunity pathways in transgenic rainbow trout harboring Cecropin P1 transgene

**DOI:** 10.1186/1471-2164-15-887

**Published:** 2014-10-11

**Authors:** Jay H Lo, Chun-Mean Lin, Maria J Chen, Thomas T Chen

**Affiliations:** Department of Molecular and Cell Biology, University of Connecticut, Storrs, CT 06269 USA

**Keywords:** Rainbow trout, Disease resistant transgenic fish, Cecropin, Antimicrobial peptide, Microarray, Innate/adaptive immunity

## Abstract

**Background:**

We have recently developed several homozygous families of transgenic rainbow trout harbouring cecropin P1 transgene. These fish exhibit resistance characteristic to infection by *Aeromonas salmonicida* and infectious hematopoietic necrosis virus (IHNV). In our earlier studies we have reported that treatment of a rainbow trout macrophage cell line (RTS11) with a linear cationic α-helical antimicrobial peptide (e.g., cecropin B) resulted in elevated levels of expression of two pro-inflammatory relevant genes (e.g., *IL-1β* and *COX-2*). Therefore, we hypothesized that in addition to the direct antimicrobial activity of cecropin P1 in the disease resistant transgenic rainbow trout, this antimicrobial peptide may also affect the expression of immune relevant genes in the host. To confirm this hypothesis, we launched a study to determine the global gene expression profiles in three immune competent organs of cecropin P1 transgenic rainbow trout by using a 44k salmonid microarray.

**Results:**

From the microarray data, a total of 2480 genes in the spleen, 3022 in the kidney, and 2102 in the liver were determined as differentially expressed genes (DEGs) in the cecropin P1 transgenic rainbow trout when compared to the non-transgenics. There were 478 DEGs in common among three tissues. Enrichment analyses conducted by two different bioinformatics tools revealed a tissue specific profile of functional pathway perturbation. Many of them were directly related to innate immune system such as phagocytosis, lysosomal processing, complement activation, antigen processing/presentation, and leukocyte migration. Perturbation of other biological functions that might contribute indirectly to host immunity was also observed.

**Conclusions:**

The gene product of cecropin P1 transgene produced in the disease resistant transgenic rainbow trout not only can kill the pathogens directly but also exert multifaceted immunomodulatory properties to boost host immunity. The identified genes involved in different pathways related to immune function are valuable indicators associated with enhanced host immunity. These genes may serve as markers for selective breeding of rainbow trout or other aquaculture important fish species bearing traits of disease resistance.

**Electronic supplementary material:**

The online version of this article (doi:10.1186/1471-2164-15-887) contains supplementary material, which is available to authorized users.

## Background

Infectious disease is one of the most severe bottlenecks in aquaculture industry. During the past few decades, different strategies including vaccination, good husbandry practice, and use of antibiotics or breeding strains less susceptible to specific diseases through conventional genetic selection have been practiced for fish disease prevention and control. These strategies, although effective to a certain extent, suffer from various drawbacks such as the high cost, labour intensiveness, emergence of antibiotics-resistant microbial strains, absence of effective cure for certain types of viral pathogens, and unsatisfactory degree of protection. Therefore, a more effective approach for controlling fish disease in aquaculture is still in high demand.

Transgenic technology promises to facilitate the genetic selection process by directly modifying the undesirable genetic traits that confer vulnerability to pathogens or introducing specific genes that are related to resistance to fish pathogens into fish [1]. The introduced transgenes can be fish-originated or characterized genes from other species. Cecropins, first identified in *Cecrpoia* moth, are members of the antimicrobial peptide (AMP) family that constitute a main part of the cell-free immunity of insects [2]. They are initially identified in a number of insect species and were later isolated from porcine small intestine, named cecropin P1 [3]. These basic peptides, 31 to 37 amino acid residues in length and consisting of mainly alpha helices, attack bacterial membrane by forming pores or ion channels and cause cell lysis [4].

An early *in vitro* study conducted in our laboratory showed that insect cecropin B and CF-17 peptide, a synthetic cecropin B analogue, effectively inhibited the replication of several fish viruses such as IHNV (infectious hematopoietic necrosis virus), VHSV (viral hemorrhagic septicemia virus), SHRV (Snakehead rhabdovirus), and IPNV (infectious pancreatic necrosis virus) [5]. Furthermore, transfer of cecropin B or cecropin P1 transgene into medaka embryos resulted in production of transgenic medaka exhibiting resistance characteristic to common fish pathogens such as *Pseudomonas fluorecens*, *Aromonas hydrophila*, and *Vibrio anguillarum* [6]. These results warrant the application of these antimicrobial peptides to commercially important fish species in order to produce disease resistant fish strains. Recently, we have successfully produced several families of transgenic rainbow trout (*Oncorhynchus mykiss*) harbouring cecropin P1 transgene under the regulation of a CMV promoter [7]. The cecropin P1 transgene is ubiquitously expressed throughout the whole body. Results of repeated challenge studies conducted on different generations of transgenic families revealed that these transgenic fish exhibited resistance characteristic to *Aeromonas salmonicida* and IHNV. Furthermore, these transgenic fish are also resistant to infection by a common rainbow trout parasite, *Ceratomyxa shasta* (Chen et al., unpublished data). In addition to the direct microbicidal activity, there is increasing evidence suggesting that AMP may exhibit a variety of immunomodulatory properties in host immune cells [8, 9]. In support of this notion, a study reported by Chiou et al. [10] from our laboratory showed that treating rainbow trout RTS11cells (a trout monocyte/macrophage cell line) with cecropin B resulted in elevation of mRNA levels of interleukin-1β (*IL-1β*), a pro-inflammatory cytokine, and cyclooxygenase-2 (*COX-2*), an enzyme crucial for the generation of inflammatory modulators. These data suggested an immunomodulatory role of cecropin B on the macrophage-regulated inflammatory response in salmonid species, yet the global effect of AMP on the expression of genes involved in innate/adaptive immunity pathways remains unstudied.

In the present study, using disease resistant transgenic rainbow trout harbouring cecropin P1 as experimental animals, we investigated the effect of AMP on global gene expression profiles at the organism level by DNA microarray analysis on a 44K oligo salmonid array platform [11]. In order to shed light on the molecular determinants essential for survival towards infectious pathogens and to unveil perturbation of biological pathways which might account for the elevated immunity of transgenic rainbow trout, we investigated three immune competent organs. RNA samples of the spleen, kidney, and liver were isolated from transgenic and non-transgenic fish and the expression datasets were analysed with two different data-mining tools. An organ specific pattern of functional pathway perturbation in cecropin P1 transgenic fish was identified. Genes of biological significance contributing to the perturbed functions were further analysed and confirmed with real-time quantitative RT-PCR (real-time RT-qPCR) analysis.

## Results

### Preliminary analysis and an overview of microarray datasets

**Figure 1:**
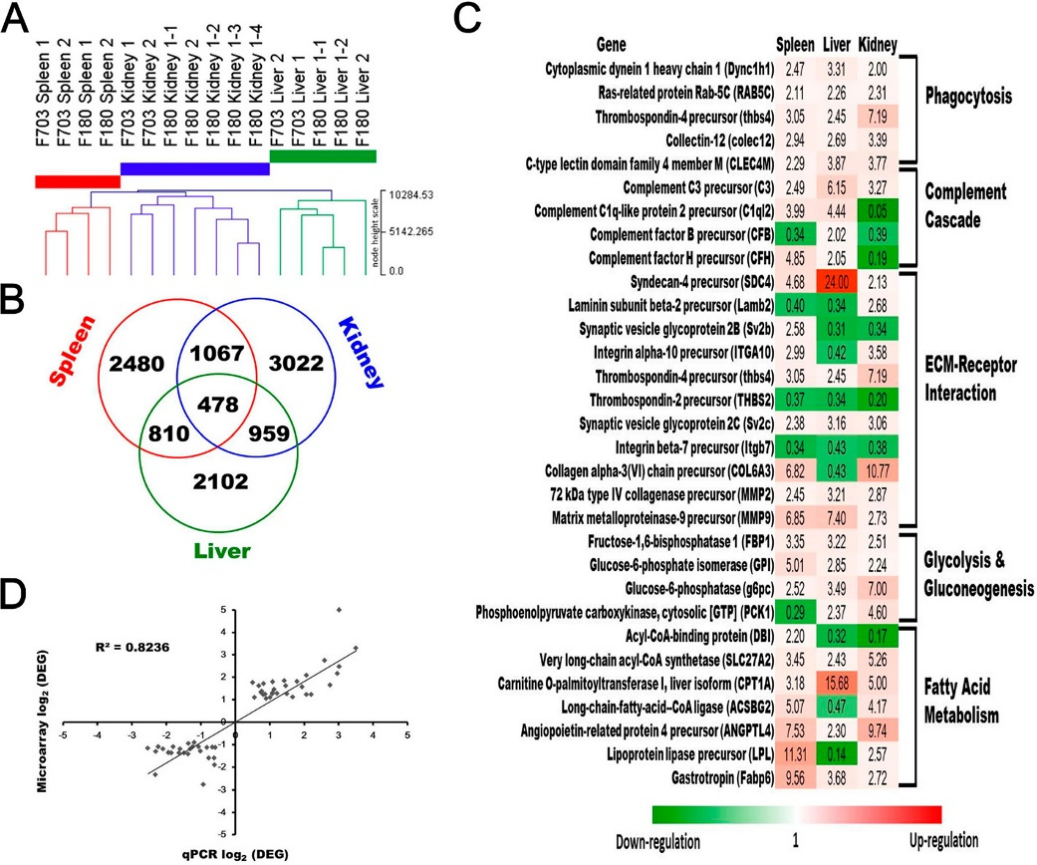
**An overview of microarray data analysed in three immune competent organs of cecropin P1 transgenic trout versus non-transgenic trout. A**, Hierarchical clustering of spleen, kidney, and liver samples derived from two families (F703 & F180) of cecropin P1 transgenic trout. Designated numbers after each tissue indicate biological and microarray technical repeats. **B**, Venn diagram showing numbers of differentially expressed genes (DEGs) identified in the three organs of cecropin P1 transgenic trout. **C**, A list of genes selected from the 478 DEGs in common among organs related to different biological functions. Color gradient denotes relative degree of expression ratio (transgenic/non-transgenic). **D**, Comparison of microarray and real-time RT-qPCR results (n = 54; linear regression, R^2^ = 0.82).

Tissue samples of spleen, kidney, and liver were collected from two different families of cecropin P1 transgenic or non-transgenic fish of equal body sizes and sexes (at one year of age). Experimental details of tissue RNA preparation, labelling of cRNA, and microarray hybridization were as described in Methods. As the initial step of preliminary analysis, normalized expression dataset was tested by Wilcoxon Rank-Sum test (p <0.05) to filter out probes reporting inconsistent signals. This generated 23563 reliable candidates in the liver and 28945 in kidney samples. Due to small number of spleen sample repeats (n = 4), only those bearing reliable signal (≥3 up- or down-regulation) were selected, which resulted in 33729 candidates. Of these candidates there were a total of 13027 probes in common among three different tissues, including those with designated gene names, those with only descriptive annotation, and those destined unknowns due to lack of homolog to any genes of other animal species based on the original probe annotation [11]. Hierarchical clustering of 16 sets of data containing these 13027 probes revealed a tissue-specific expression profile. There was a shorter distance between sample clusters of spleen and kidney as opposed to that of liver (Figure 1A). The clustering of samples of the same tissue suggested limited biological and technical variations, indicating data consistency. A closer expression profile between spleen and kidney versus that of liver was in line with their similar roles in terms of immune function since kidney and spleen are the primary and secondary immune organs in teleost fish [12]. To further refine the data, the candidates with expression ratio over two-fold (transgenic/non-transgenic >2; <0.5) were gated and defined as differentially expressed genes (DEGs). Elimination of redundancy (probes annotated with identical gene name) and unknowns reduced the numbers of DEG to 2480 in the spleen, 2102 in the liver, and 3022 in the kidney. There were 478 DEGs in common among three tissues (Figure 1B; Additional file 1 for complete gene list). Genes selected from these DEGs implicated in different biological functions show consistent or inverse expression pattern among tissues (Figure 1C). To evaluate gene expression values obtained by microarray analysis, selected genes covering a diverse range of expression ratios were confirmed with real-time RT-qPCR analysis. Comparison of these two independent methods revealed a general agreement and a high degree of linear correlation (R^2^ = 0.82, Figure 1D), indicating the expression dataset was sound.

### Enrichment of biological terms of Gene Ontology (GO) and KEGG pathway by GeneCodis

**Figure 2:**
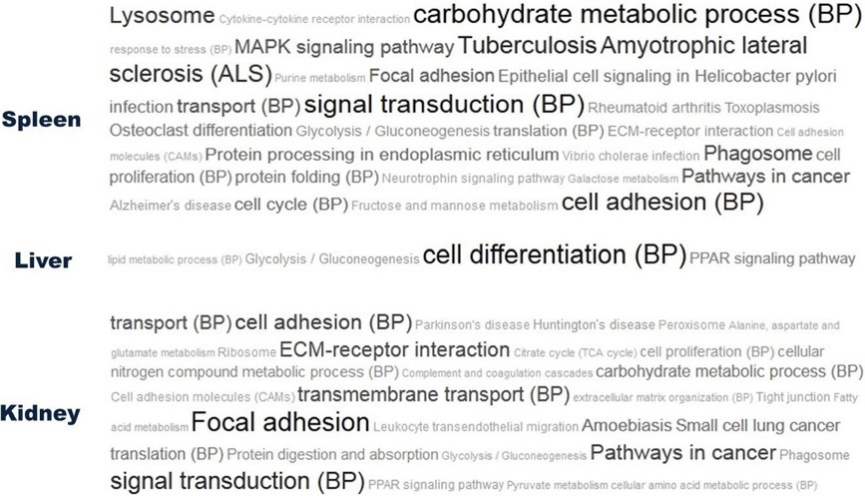
**Tag cloud visualization of biological terms enriched by GeneCodis modular enrichment analysis in different organs of cecropin p1 transgenic trout.** Cloud of tags containing the most significant 30 terms derived from either KEGG or GO Biological Process (BP) databases was displayed for each tissue. The tag’s sizes vary according to the number of supporting gene.

As the first step toward uncovering thematic association of gene expression change in cecropin P1transgenic fish, we conducted enrichment analysis by adopting GeneCodis. GeneCodis is an over-representation analysis (ORA) approach that can perform both singular and modular enrichment analyses via retrieving biological terms from different databases [13, 14]. The modular enrichment analysis integrates heterogeneous annotations and discovers significant combinations among them. Since different databases collect distinct biological terms annotated with similar or dissimilar collection of genes, applying modular enrichment analysis improves discovery sensitivity and specificity [15]. DEGs of each tissue and the total genes present on the 44k microarray chip serving as a reference gene list were used as input to inquire GOSlim Process and KEGG pathway databases. Tag clouds displaying the most prominent terms showed distinct patterns of enrichment (Figure 2). A lot more biological terms were identified in the spleen and kidney than in the liver. Ranking these terms in order of statistical significance (Table 1) revealed that in the spleen, biological functions including lysosome, phagosome, cell adhesion, and basic metabolic function (carbohydrate, amino sugar, and nucleotide sugar) were most significantly impacted. In the kidney, however, biological functions related to cell-cell interaction and cell movement, such as ECM-receptor interaction, focal adhesion, and amoebiasis were perturbed. In addition, translational machinery was also affected. In the liver, only four biological terms were enriched and three of them including PPAR signalling were associated with lipid and sugar metabolism. These biological terms were also present among the most significantly enriched terms reported by singular enrichment analysis (Additional file 2). Examining a complete list (p <0.05) revealed enrichment of terms related directly to immune system, such as antigen processing and presentation in the spleen and leukocyte transendothelial migration in the kidney (Additional file 2, KEGG term). Additionally, significant enrichment of carbohydrate metabolic process (Additional file 2, GO term) in the spleen and kidney suggested the possibility of a global perturbation of basic metabolic function in the transgenic fish.

**Table 1:** **Co‒occurrence annotations of GO and KEGG terms found by GeneCodis**

Biological terms	Genes (I/R) ^1^	P value ^2^	Identifier
**Spleen**			
Lysosome	45/106	7.68E-08	KEGG: 04142
Carbohydrate metabolic process	66/228	1.71E-04	GO: 0005975
Tuberculosis	15/25	2.04E-04	KEGG: 05152
Phagosome			KEGG: 04145
Phagosome	32/82	2.26E-04	KEGG: 04145
Cell adhesion	91/353	2.49E-04	GO: 0007155
Lysosome	10/13	3.69E-04	KEGG: 04142
Tuberculosis			KEGG: 05152
Lysosome	10/14	9.47E-04	KEGG: 04142
Phagosome			KEGG: 04145
Lysosome	9/12	1.20E-03	KEGG: 04142
Rheumatoid arthritis			KEGG: 05323
Lysosome	9/12	1.20E-03	KEGG: 04142
Tuberculosis			KEGG: 05152
Phagosome			KEGG: 04145
Amino sugar and nucleotide sugar metabolism	19/43	1.28E-03	KEGG: 00520
**Liver**			
Glycolysis/Gluconeogenesis	17/43	7.76E-03	KEGG: 00010
Cell differentiation	71/322	9.78E-03	GO: 0030154
PPAR signaling pathway	17/47	1.98E-02	KEGG: 03320
Lipid metabolic process	9/17	2.63E-02	GO: 0006629
PPAR signaling pathway			KEGG: 03320
**Kidney**			
Focal adhesion	29/47	2.72E-07	KEGG: 04510
ECM-receptor interaction			KEGG: 04512
Protein digestion and absorption	31/56	9.63E-07	KEGG: 04974
ECM-receptor interaction	33/61	9.76E-07	KEGG: 04512
PPAR signaling pathway	27/47	2.66E-06	KEGG: 03320
Protein digestion and absorption	12/13	7.68E-06	KEGG: 04974
Focal adhesion			KEGG: 04510
ECM-receptor interaction			KEGG: 04512
Focal adhesion	15/21	7.04E-05	KEGG: 04510
Amoebiasis			KEGG: 05146
ECM-receptor interaction			KEGG: 04512
Signal transduction	196/743	1.58E-04	GO: 0007165
Translation	71/216	2.34E-04	GO: 0006412
Focal adhesion	18/31	3.09E-04	KEGG: 04510
Cell adhesion			GO: 0007155
ECM-receptor interaction			KEGG: 04512
Protein digestion and absorption	9/10	4.16E-04	KEGG: 04974
Focal adhesion			KEGG: 04510
Amoebiasis			KEGG: 05146
ECM-receptor interaction			KEGG: 04512

For spleen and kidney, only the first 10 most significant groups of terms were listed. ^1^I/R, Number of annotated genes in the input list/number of annotated genes in the reference list; ^2^P values were obtained through hypergeometric analysis and adjusted for multiple corrections using FDR.

### Gene set enrichment analysis

The enrichment analysis conducted by GeneCodis used pre-defined (>2 fold) DEGs as input, which exclude genes of minor fold changes that could be of biological significance in a given pathway. Gene set enrichment analysis (GSEA) evaluates microarray data at the level of gene sets by taking all genes from a microarray experiment without applying a cut-off. It reduces arbitrary factors and allows minimally changing genes, which cannot pass the selection threshold, to contribute to the enrichment analysis in differing degrees [16]. To perform GSEA, non-redundant genes bearing reliable signal on the 44k platform (10875 in the spleen, 8882 in the liver, and 9970 in the kidney) were ranked in order of expression ratios followed by GSEA using gene set definition of KEGG and GO Biological Process.

**Figure 3:**
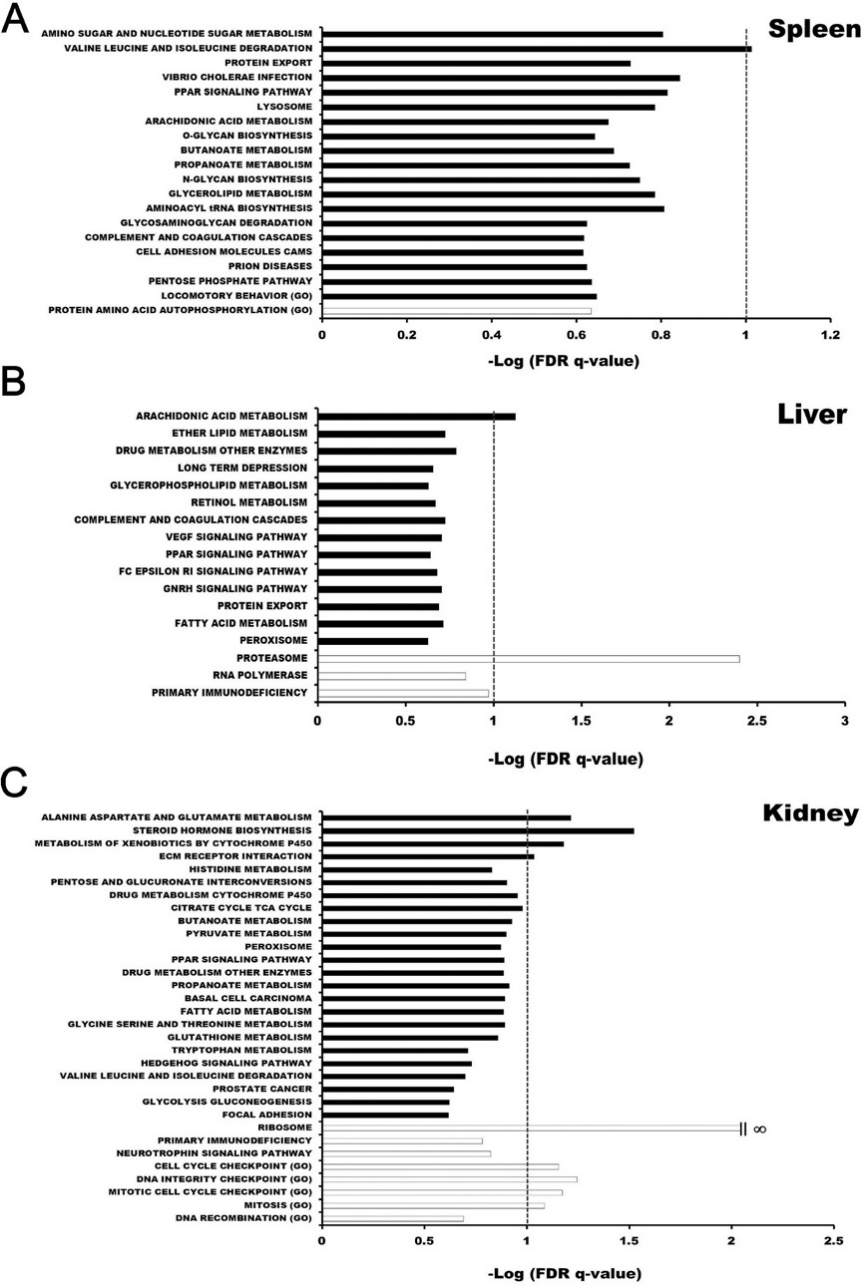
**Perturbation of biological functions and pathways reported by GSEA in different organs of cecropin P1 transgenic trout. A**, Spleen; **B**, Liver; **C**, Kidney. The graph shows the negative log FDR q values of the enriched gene sets reported by GSEA. Gene sets (nominal p value <0.05; FDR q value <25%) were ordered, from top to bottom, by normalized enrichment scores (NES) (closed bar, positive NES, suggesting up-regulation; open bar, negative NES, suggesting down-regulation). The crossing line indicates statistical significance cut-off (FDR q value =0.1). Gene sets derived from GO Biological Process database were denoted (GO).

Combining results obtained from two databases, there were a total of 20 minimally qualified gene sets enriched in the spleen, 32 in the kidney, and 17 in the liver (nominal p <0.05; FDR < 0.25, Figure 3A-C). Gating false discovery rate (FDR) to 10% revealed that in the spleen there was only one gene set, valine, leucine, and isoleucine degradation, reaching the threshold. In the liver, proteasomal pathway was enriched with a negative enrichment score and highest probability (FDR = 0.4%), implying a decreased rate of serum protein turnover in the transgenic trout. Additionally, arachidonic acid metabolism, a biochemical process metabolizing phospholipids of cell membrane, was the only up-regulated gene set passing the threshold. The result reinforced validity of the similar detection, lipid metabolic process, by GeneCodis (Table 1). In the kidney, there were nine gene sets passing 10% FDR, including four up-regulated and five down-regulated. Among the four up-regulated gene sets, ECM receptor interaction as well as alanine, aspartate, and glutamate metabolism were corroborated by previous analysis (Table 1; Additional file 2), whereas steroid hormone biosynthesis and metabolism of xenobiotic were newly identified. Interestingly, ribosomal protein was the most significantly enriched gene set in the kidney (nominal P = 0, FDR = 0) and it was down-regulated. This was in line with the detection by GeneCodis analysis (Table 1; Additional file 2) and suggesting the suppression of translational machinery in the kidney. The other four down-regulated gene sets were related with cell cycle and cell division, such as DNA integrity checkpoint and mitosis. While not passing the 10% FDR threshold, a couple of marginally qualified gene sets supported the results of GeneCodis analysis, such as lysosome as well as amino sugar and nucleotide sugar metabolism in the spleen, fatty acid metabolism and PPAR signalling in the liver, as well as focal adhesion in the kidney. Therefore, these biological functions could likely be perturbed also in the corresponding tissues of cecropin P1 transgenic trout.

### Altered expression of genes of biological importance in cecropin P1 transgenic rainbow trout

**Figure 4:**
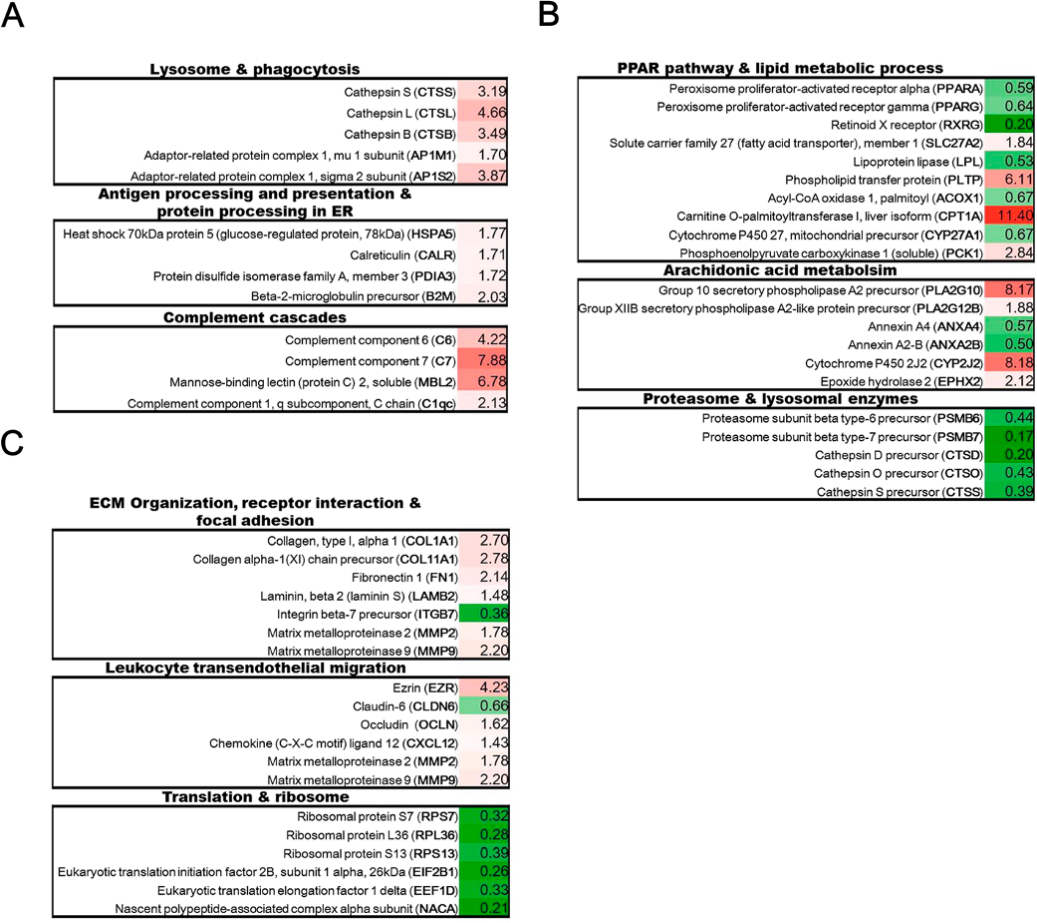
**Altered expression of genes implicated in the perturbed biological functions of cecropin P1 transgenic trout. A**, Spleen; **B**, Liver; **C**, Kidney. Expression of selected genes of functional importance involved in different biological pathways identified by GeneCodis and GSEA was analyzed and confirmed with real-time RT-qPCR. Color gradient denotes relative degree of expression ratio (transgenic/non-transgenic).

Enrichment analyses have so far identified perturbation of biological functions in different organs of the transgenic trout. To gain further insight into members contributing to the enrichments, we examined genes within individual biological term and selected those of functional importance for confirmation by real-time RT-qPCR analysis (Figure 4A-C). A complete list of genes with expression values contributing to individual term that was either statistically most significant or relevant to immune function was presented in Additional file 3. In the spleen of transgenic trout, several genes related to lysosomal function and phagocytosis were up-regulated. This included multiple isoforms of cathepsin, a lysosomal protease participating in the degradation of antigenic proteins, and adaptor-related protein complex which links clathrin to sorting receptors in coated vesicles for trafficking hydrolytic enzyme from Golgi to endosome. Phagocytosis and lysosomal digestion were tightly linked to antigen processing and presentation. Indeed, ER-localized chaperones including HSPA5, PDIA3, and calreticulin, which modulate proper folding of MHC class I and its association with β2 microglobulin light chain, were induced. Genes of complement system were also significantly enriched. This included the up-regulation of C6 and C7, which were membrane attack complex (MAC) components, as well as C1q and mannose-binding lectin, which were initiators of classical and lectin pathway, respectively. In the kidney, ECM turnover was activated. Such detection included the up-regulation of multiple types of collagen, fibronectin, and matrix metalloproteinase (MMP). Besides, perturbed expression of integrin was also observed. This suggested perturbation of cell–matrix interaction and a possible impact on leukocyte mobilization. Indeed, ezrin, a cell membrane-cytoskeleton linker and tyrosine kinase substrate, playing a key role in the regulation of cell adhesion and migration, were up-regulated. Other affected genes included chemokine ligand 12 (CXCL12), which is strongly chemotactic for lymphocytes, claudin and occludin, which are components of the epithelial cell tight junctions, as well as MMP-2 and -9, which are critically involved in ECM degradation and leukocyte transendothelial migration. Interestingly, a noticeable amount of ribosomal protein subunits and regulatory molecules of translational machinery were down-regulated, indicating retardation of de novo protein synthesis. In the liver, PPAR signalling was supressed, including the down-regulation of PPAR-α, -γ and the heterodimerization partner, RXR-γ. PPAR target genes or the others regulating lipid transport, fatty acid oxidization, and cholesterol metabolism were either up- or down-regulated, suggesting perturbation of energy metabolism. Arachidonic acid is a polyunsaturated fatty acid abundant in the liver. It is freed from phospholipids of cell membrane by phospholipase A2 and subsequently converted by various enzymes into signalling molecules, eicosanoids, exerting complex control over inflammation or immunity. The up-regulation of several corresponding enzymes implicated in the metabolic pathway and the down-regulation of phospholipase A2 inhibitor, annexin, implied an increased production of inflammatory modulators. Lastly, multiple subunits of proteasomal complexes and cathepsin were confirmed down-regulated, indicating a decreased proteolytic activity in the liver.

## Discussion

Cationic AMPs, such as cecropin P1, were well-recognized for the direct cytotoxic activities toward bacteria and viruses as their primary function in host defence [17]. Accumulated evidence from recent studies in mammals, however, has indicated that AMPs may exhibit multifaceted immunomodulatory properties via altering gene expression in a variety of host cells [8, 9]. Such immunomodulatory effect on different targets can cause altered expression of chemokine and cytokine, activation of leukocyte, stimulation of angiogenesis, promotion of wound healing, and the others. These distinct biological activities exerting on invading pathogens and the host ensure rapid clearance and resolution of infection and repair of damaged epithelia [8]. We have developed cecropin P1 transgenic rainbow trout and demonstrated their enhanced resistance to bacterial and viral pathogens compared to the non-transgenics [7]. Therefore, these transgenic fish are ideal experimental animals for addressing the question of how cecropin P1 modulates host immunity or other genetic traits and confers them resistant to infection by pathogens. By adopting the 44k salmonid microarray and analysing expression datasets with different data-mining tools and databases, we uncovered perturbation of gene clusters controlling distinct biological processes in three immune competent organs. While there is no unified standard for analysing high-throughput transcriptome data at present, adopting multiple analytical tools using different algorithms and searching different databases collecting overlapping or unique biological terms may provide confidence and complementation in generating hypothesis [14, 16]. By combining all information in the current study, we have generalized major functional perturbations and molecular events taken place in spleen, kidney, and liver of the transgenic fish. Herein we discuss the conceivable correlations with pathogen-resistant characteristic of the transgenic fish in light of knowledge in the literature.

### Spleen

Similar to that in higher vertebrates, spleen is one of the main lymphoid organs in teleost fish. The spleen of teleost fish is composed of a system of splenic ellipsoid, melano-macrophage centres (MMCs), and lymphoid tissue. Such structural complexity can facilitate blood filtering and augment efficiency in trapping and processing antigens [12, 18, 19]. Histologically, the MMCs exist as discrete aggregates of pigment-containing cells consisting primarily of macrophages and lymphocytes. They are structurally and functionally similar to the germinal centres of lymph nodes in mammals, which facilitate antigen trapping and provide sites for mature lymphocyte to interact with antigens [20]. The splenic ellipsoids are terminal branches of arterioles bearing thickened walls where blood-borne antigens and immune complexes may be detained for long periods of time. This environment allows phagocytic cells along the walls to act as sessile or fixed cells and promotes effective ingestion and presentation of antigens to lymphocytes, which is essential for the initiation of adaptive immune response and establishment of immunological memory [12, 21]. Interestingly, the activation of multiple interrelated pathways in the spleen conforms to its major immunological function, the centre of mononuclear phagocyte system. The up-regulated expression of multiple genes including lysosomal hydrolases, vacuolar ATP-dependent proton pump mediating acidic pH of lysosomal compartment, and factors involved in shuffling enzymes from Golgi to endosome or regulating vesicle traffic from late endosome to lysosome, that we observed in this study, strongly indicated an enhanced activity of digesting internalized substances. The complement system is one arm of non-specific humoral defence playing diverse roles including foreign cells lysis, opsonisation, activation of inflammatory response, and immune complex clearance. Teleost fish possess well-developed complement system resembling that of mammals [22]. Rainbow trout complement can inactivate toxic extracellular products of *A. salmonicida* [23], and it has been demonstrated that salmon macrophages possess complement receptors that bind C3, the major phagocytosis promoting factor [24, 25]. The increased levels of C3 and other key components of the complement system, such as C7and MBL2, can therefore promote bactericidal activity and pathogen uptake. The processed antigens in the lumen of phagocytic or endocytic organelles are loaded onto MHC class II molecules followed by translocation onto cell surface for presentation to CD4^+^ T cells. Whereas this is the canonical pathway for the presentation of exogenous antigens, studies have indicated that phagocytosed antigens can also be presented by MHC class I molecules, known as cross-presentation [26]. This process involves likely the utilization of ER-associated degradation (ERAD) machinery, evidenced by the presence of ER-resident proteins on phagosome due to ER-phagosome fusion, to mediate translocation of partially digested microbial proteins from the phagosome lumen to cytosol where antigens are ubiquinated and further processed by proteasome. The processed small peptides are then transported into ER lumen by transporter for antigen processing (TAP) complex, loaded onto MHC-I molecule, and subsequently exported via classical secretory pathway to the cell membrane [27]. A concerted induction of a large body of candidate molecules critically involved in this series of processes was identified in the current study. This included subunits of SEC61 complex, responsible for exporting misfolded protein across ER membrane and facilitating protein translocation from phagosome lumen to cytosol, heat shock proteins (HSP90) involved in the degradation of cytosolic antigen by proteasome, tapasin (TAPBP), mediating interaction between newly assembled MHC-I and TAP, as well as ER chaperones (HSPA5/BiP, PDIA3, and CALR) required for protein quality control and loading of peptide onto MHC-I. Although the details of cross presentation in teleost fish await study, it is known that cellular constituents responsible for antigen processing and presentation are conserved from teleost to mammal [28, 29]. Accordingly, the up–regulation of multiple genes involved in this process, particularly the MHC-I pathway, suggested an enhanced ability to neutralize virus-infected host cells. This was supported by the demonstrated resistance of these transgenic fish to viral infection. As a whole, the enhancement of these coherent processes in the spleen of cecropin P1 transgenic trout could coordinately contribute to accelerating pathogen clearance and increasing the capacity of antigen presentation to T lymphocytes for mounting adaptive immune responses.

### Kidney

Teleost fish lack bone marrow and the anterior (head) kidney is the largest site assuming haematopoietic function equivalent to that of bone marrow in higher vertebrates. The teleost kidney is the primary lymphoid organ where B lymphocytes are produced and matured and houses many myeloid lineage cells such as granulocytes and monocytes/macrophages, providing the first line of non-specific defence. Like the spleen, MMCs are also present in the kidney of teleost fish. Therefore, it also serves as a secondary lymphoid organ where mature lymphocytes interact with antigens and are activated to establish long-lived specific immunity [12, 18, 19]. In this study we found three major perturbations in the kidney of transgenic fish: 1) altered ECM organization and leukocyte-ECM interaction; 2) suppressed translational machinery; and 3) plausibly, perturbed cell cycle progression and mitosis. It was shown that trout kidney harbors B cells of different developmental stages in accordance with tissue polarity [30]. The progenitor B cells mature and become antigen-responsive at the anterior side. They then migrate to the spleen via circulation or within the kidney to the posterior side where mature B cells are activated by antigen presenting- and T- cells, resulting in differentiation into plasmablasts and eventually plasma cells. Subset of these cells will subsequently home back to the head kidney, which forms reservoir maintaining abundant long-lived antibody-secreting plasma cells. Such homing behaviour mirrors that in tetrapod bone marrow in which environmental cues derived from surrounding stromal cells and ECM components provide supportive niches that sustain plasma cell long-term survival [31–33]. Although the degree of analogy in terms of molecular cues and cell type composition responsible for the comparable functions between teleost head kidney and tetrapod bone marrow needs further characterization, cell culture developed from trout pronephros showed morphological characteristics similar to the mammalian counterparts and supported the development of haematopoiesis when co-cultured with haematopoietic precursors [34, 35], implying the corresponding molecular and cellular bases might be conserved. The ECM has an important function in modulating immune cell behaviours such as cell proliferation, differentiation and survival, intercellular communication, and leukocyte trafficking into inflamed tissues. Although the role of individual ECM component in maintaining bone marrow niches has not been precisely defined, dynamic interaction of adhesion molecules, growth factors, and chemokines with receptors on cell surface are involved in lodging and homing of hematopoietic progenitors and effector B cells [31, 33, 36, 37]. In this respect, significant altered expression of multiple types of integrin, ECM components, as well as enzymes (MMP) controlling ECM turnover/remodelling could signify a fundamentally different microenvironment in the kidney of transgenic trout which favours immune cell maturation, activation, or survival in order to maintain sustained titer of systemic antibodies.

Tissue inflammation is commonly accompanied by leukocyte influx and ECM remodelling [38]. In gilthead seabream, the expression of several ECM-related molecules responds to pro-inflammatory stimuli in immune competent tissues [39]. In the same species, it was also shown that collagen and gelatin can induce intracellular accumulation of IL-1β and prime respiratory burst of phagocytes isolated from the head kidney, indicating that collagen fragments produced by the action of different host proteases (MMPs) are sensed by phagocytes in teleost fish [40]. The respiratory burst generates reactive oxygen species (ROS) which has potent microbicidal activity. Besides, although the mechanism has not been fully elucidated, several studies indicated that ROS can inhibit mRNA translation [41–44]. The generation of ROS is commonly tied to NLRP3 inflammasome activation in response to a variety of agonists, which ultimately leads to proteolytical processing of inflammatory cytokines, IL-1β and IL-18, by the activated caspase-1 [45–47]. Inflammasome-mediated activation of caspase-1 also triggers programmed lytic cell death termed pyroptosis, a process frequently induced by infection and results in cell burst releasing cytosolic contents such as cytokines or recalcitrant pathogens. Pyroptosis augments inflammation and exposes pathogens to uptake and killing by neutrophils [48, 49]. A study in seabream leukocytes has revealed that infection of macrophage leads to caspase-1 independent processing and release of IL-1β as well as caspase-1 dependent pyroptotic cell death, suggesting a role for inflammasome and caspase-1 in the clearance of infected immune cells in teleost fish [50]. Furthermore, a recent study using bone marrow-derived macrophages showed that inhibition of translation per se is sufficient to trigger NLRP3 inflammasome activation [51]. It is therefore tempting to speculate that the kidney of transgenic trout is under a status of chronic inflammation with the acquisition of enhanced ability to restrict proliferation and promote clearance of invading pathogens.

Cell proliferation is regulated by a variety of signals. The perturbation of cell cycle progression might be related to the aforementioned events such as ROS production, release and activation of extracellular factors, or alteration of cell contacts as a consequence of ECM turnover. Reducing protein synthesis might also control proliferation as cell cycle arrest is a well-established consequence of the general translational arrest.

### Liver

The vertebrate liver is an essential immune organ in which cellular immune functions are met predominantly by natural killer cells, CD8^+^ T cells, and scavenging phagocytes [52]. The liver carries out many processes related to energy metabolism and is also a metabolic centre for protein synthesis and degradation. As in mammals, teleost liver is the major production site of a number of plasma proteins including acute-phase protein, complement component, serum albumin, apolipoprotein, lysozyme, and AMP. These protein factors constitute parts of the humoral immunity [18, 53, 54]. The intracellular degradation of protein can be achieved by lysosomal enzyme dependent proteolysis or ubiquitin-proteasome pathway. Interestingly, the suppressed expression of numerous genes encoding proteasomal subunits and lysosomal proteases in the transgenic trout suggested a reduced protein turnover. This phenomenon might be beneficial in maintaining sustainable reservoir of humoral factors for swift neutralization of invading pathogens. Decreased rate of protein degradation also reduces the energy cost for de novo protein synthesis. Indeed, mounting an immune response is an energy demanding process, which has effects in many physiological pathways in the body. Several studies utilizing high throughput techniques have revealed altered transcriptomic profiles and pathogen resistance in response to starvation and feeding regimen, particularly the fatty acid composition in diet, in the liver or immune cell line of salmonids, indicating a strong linkage between energy metabolism and immune function [55–59]. Correspondingly, numerous gene clusters related to lipid, amino acid, and sugar metabolism were perturbed in the liver and were also in the spleen and kidney of transgenic trout. Fatty acids are used as an energy source and structural components of cell membrane. They also serve as precursors for the synthesis of eicosanoids, which are immuomodulatory signalling molecules [60–62]. In the liver of transgenic fish, up-regulated expression of phospholipase A2, which releases fatty acids from phospholipid membrane, and the down-regulation of its inhibitors, annexin A2 and A4, could contribute to an increased production of arachidonic acid. However, perturbed expression of other enzymes implicated in processing the downstream metabolites seems to promote production of prostaglandins, thromboxanes, and lipoxins but disfavour the production of leukotrienes and prostacyclins. As these eicosanoids exert complex immune regulation of both pro- and anti-inflammatory properties and can also stimulate or inhibit leukocyte proliferation [62–64], it is difficult to predict their overall impact on the immunity of the transgenic trout. Eicosanoids are ligands that can activate PPAR signalling and modulate PPAR targeting. PPARs are ligand-activated nuclear receptors which form obligate heterodimers with RXR and interact with specific DNA sequence to regulate target gene expression. In mammals, PPARs act as lipid sensor by binding with endogenous fatty acid metabolites and regulate genes predominately associated with lipid metabolism. Additionally, abundant evidence suggests that PPARs, specifically PPAR-α and -γ, are regulators of immune functions. Ligands targeting these receptors have therapeutic activity in autoimmune and inflammatory diseases [65–67]. In teleost, sporadic studies have suggested that fish PPAR-α, similar to its mammalian counterpart, is strongly activated by a range of unsaturated fatty acids. However, fish PPAR-γ is not effectively activated by fatty acids or mammalian PPAR-γ specific agonists, suggesting the potential functional divergence between fish and mammal PPAR-γs [68, 69]. At present, little is known about endogenous PPAR ligands in fish and functions of salmonid PPARs are further complicated with the existence of multiple subtypes derived from transcript variants in each isoform and the polyploid genome of salmonids [69, 70]. Nevertheless, perturbed expression of numerous PPAR target genes controlling fatty acid oxidization and transport, cholesterol metabolism, adipocyte differentiation, and gluconeogenesis suggested a systemic alteration of lipid homeostasis. Taken together, these physiological changes in the liver of transgenic trout indicate a reallocation of body energy reserves for mounting a constitutive immune response.

In the current study, we have identified a number of biological pathways in each organ that could potentially contribute to the enhanced immunity and disease resistance of cecropin P1 transgenic rainbow trout. Further detailed functional analyses are required to prove these hypotheses. The microarray chip used in this study is currently the most updated platform with comprehensive transcript representation for salmonid species. Given the availability of RNA sequencing (RNA-seq), it will be of great merit to profile the complete transcriptome in the future and identify key genes closely associated with pathogen resistance. These genes can serve as molecular marker for marker-assisted selective breeding for salmonids or other aquaculture important fish species. A similar approach involving the development of single nucleotide polymorphism (SNP) microarray for selective breeding of Atlantic salmon has recently been reported [71].

## Conclusions

In the present study, we have identified multiple functional perturbations in the spleen, kidney, and liver of cecropin P1 transgenic trout. Some of the perturbed biological pathways are related directly to innate/adaptive immune functions, while the others might contribute indirectly to enhancing host immunity. The present study is the first example that a true disease resistant fish is used as experimental animals for functional genomic analysis and the results corroborate existing knowledge of the multifaceted immunomodulatory property of cationic host defence peptide. The identified genes in this study may serve as useful markers for selective breeding of salmonids or other aquaculture important fish species bearing traits of pathogen resistance.

## Methods

### RNA preparation

A total of thirteen RNA samples were prepared from the three tissues, namely liver, kidney, and spleen of transgenic and non-transgenic rainbow trout (one year of age) maintained in the Salmon Disease Laboratory at Oregon State University (OSU protocol # 4282). Tissue samples were collected from two individuals of two transgenic and one non-transgenic rainbow trout families. For both microarray and real-time RT-qPCR analyses, total RNA was isolated from freshly dissected tissue samples using TRIzol reagent (15596–018, Life Technologies, Carlsbad, CA) following the protocol provided by the manufacturer. All of the RNA samples were treated with DNase-I (M610A, Promega, Madison, WI) to remove contaminating genomic DNA and followed by TRIzol extraction. RNA quality and concentration was determined on Agilent Bioanalyzer (Agilent Technologies, Santa Clara, CA) and NanoDrop spectrophotometer (NanoDrop Technologies, Wilmington, DE).

### cRNA synthesis, labelling, and microarray hybridization

The following procedures were conducted by Ambry Genetics (Aliso Viejo, CA). Briefly, two-color labeling reactions (300 ng-3 μg total RNA as input) were prepared by using Agilent Two-Color Quick Amp Labeling kit (version 6.0, Agilent Technologies) following manufacturer’s protocol. First, 300 ng-3 μg of total RNA with the appropriate concentration of RNA spike-in controls was converted into double stranded cDNA using an oligo (dT) primer linked to the T7 promoter sequence and MMLV-RT. The double stranded cDNA was then *in vitro* transcribed by T7 RNA polymerase, which simultaneously amplified and incorporated cyanine 3-labeled or cyanine 5-labeled CTP into the resulting cRNA. Labeled cRNA was then purified using Qiagen RNeasy columns (74104, Qiagen, Hilden, Germany) and the labeling efficiency determined by NanoDrop spectrophotometer. Next, equal amounts of cyanine 3- and cyanine 5-labeled cRNAs were fragmented and placed on the custom 44K salmonid microarray for hybridization carried out at 65°C for ~17 hours. Finally, the arrays were washed and scanned at 5 μm resolution on an Agilent G2565CA High Resolution Scanner (Agilent Technologies).

### Reverse transcription (RT) real-time PCR (qPCR) analysis

Two μg of DNase-treated RNA samples were converted into first strand cDNA by using Superscript III reverse transcriptase (18080–044, Life Technologies) and oligo (dT)17 following conditions provided by the manufacturer. The synthesized cDNA samples were brought up to a final volume of 100 μl by DNase-free water. For qPCR assay, amplifications were carried out in a 96 well plate by C1000 thermal cycler/CFX96 Real-Time PCR Detection System (Bio-Rad, Hercules, CA). Each cDNA sample was analysed in triplicate in 20 μl final reaction volume containing 1 μl cDNA, 1x SsoFast EvaGreen Supermix (172–5201, Bio-Rad), 0.01 μM fluorescein (170–8780, Bio-Rad), and 0.5 μM gene specific primers (Additional file 4). The thermal cycle consists of an initial denaturation for 2 min at 98°C followed by 40 cycles of 5 sec at 98°C and 30 sec at 59°C in which the machine read the plate. A melting curve, from 65°C to 95°C with 0.5°C increments every 5 sec, was added after each amplification run for quality control. Cytochrome c-1 (CYC1), present in the microarray platform and did not show differences in expression between tissue samples of the transgenic and non-transgenic fish, was used as a reference gene. The threshold cycle (Ct) values were retrieved by CFX manager (Bio-Rad) and analysed by Q-Gene [72] to determine differential expression (transgenic/ non-transgenic).

### Bioinformatics analysis of expression dataset

Hierarchical Clustering (HCL) was performed by MultiExperiment Viewer (MeV, v4.9). A total of 16 sets of data comprising 13027 expression values were subjected to HCL using Manhattan distance metric and average linkage clustering to construct the hierarchical tree and determine cluster-to-cluster distances. For enrichment analysis performed by GeneCodis (http://genecodis.cnb.csic.es/), the pre-defined DEGs of each tissue (Figure 1B) and total genes present on the 44k microarray chip serving as a reference gene list were used as input to inquire GOSlim Process and KEGG pathways. The default statistical parameters (minimum number of genes: 3; statistical test: hypergeometric; p-value correction: FDR) were applied for both singular and modular enrichment analyses. In GSEA, the median expression values of sample repeats and redundant probes were used to determine the final expression value of each gene. The pre-ranked gene list and the custom 44k chip annotation file were used as input to inquire KEGG and GO BP gene sets (Molecular Signatures Database: c2.cp.kegg.v4.0; c5.bp.v4.0). The GSEA Java desktop program was downloaded from http://www.broadinstitute.org/gsea/index.jsp and the default parameters of GSEA Pre-Ranked module (number of permutations: 1000; enrichment statistic: weighted; normalization mode: meandiv; seed for permutation: timestamp; 15 < gene set size <500) were applied for analyses.

## Electronic supplementary material

Additional file 1: **A complete list of common DEGs of the three organs.** (XLSX 47 KB)

Additional file 2: **Complete lists of biological terms in each organ reported by GeneCodis singular enrichment analysis.** (XLSX 46 KB)

Additional file 3: **Functional gene clusters of importance and significance in each organ reported by GeneCodis and GSEA.** (XLSX 37 KB)

Additional file 4: **Primers for real-time RT-qPCR.** (XLSX 15 KB)
